# Supplementary material for: GDAP1 loss of function inhibits the mitochondrial pyruvate dehydrogenase complex by altering the actin cytoskeleton
Source: Commun Biol. 2022 Jun 3;5:541. doi: 10.1038/s42003-022-03487-6 (PMC9166793; doi:10.1038/s42003-022-03487-6)
Supplement: Supplementary file 1 — Supplementary Information [file 42003_2022_3487_MOESM1_ESM.pdf]

GDAP1 loss of function inhibits the mitochondrial pyruvate dehydrogenase complex by altering the actin cytoskeleton

Christina Wolf[1], Alireza Pouya[1], Sara Bitar[1], Annika Pfeiffer[1], Diones Bueno[1], Liliana Rojas-Charry[1], Sabine Arndt[2], David Gomez-Zepeda[2], Stefan Tenzer[2], Federica Dal Bello[1,3], Caterina Vianello[1,3], Sandra Ritz[5], Jonas Schwirz[5], Kristina Dobrindt[6], Michael Peitz[6,7], Eva-Maria Hanschmann[8], Pauline Mencke[9], Ibrahim Boussaad[9,10], Marion Silies[11], Oliver Brüstle[6], Marta Giacomello[3,4], Rejko Krüger[9,12], and Axel Methner[1#]

Supplemental figure 1

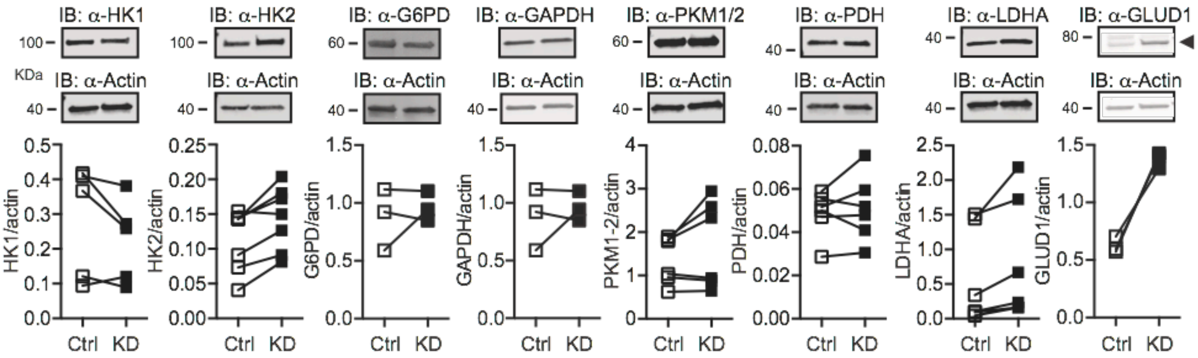

Supplemental Figure 1 Changes in protein abundance. (a) Immunoblot of whole cell lysates from Ctrl and GDAP1 KD cells against Hexokinase 1 (HK1), HK2, Glucose-6-phosphate dehydrogenase (G6PD), Glyceraldehyde 3-phosphate dehydrogenase (GAPDH), Pyruvate kinase M1/2 (PKM1/2), Pyruvate dehydrogenase (PDH), Lactate Dehydrogenase A (LDHA), Glutamate dehydrogenase (GLUD1, arrowhead). Sizes are indicated and actin served as loading control. n=3-7

Supplemental figure 2

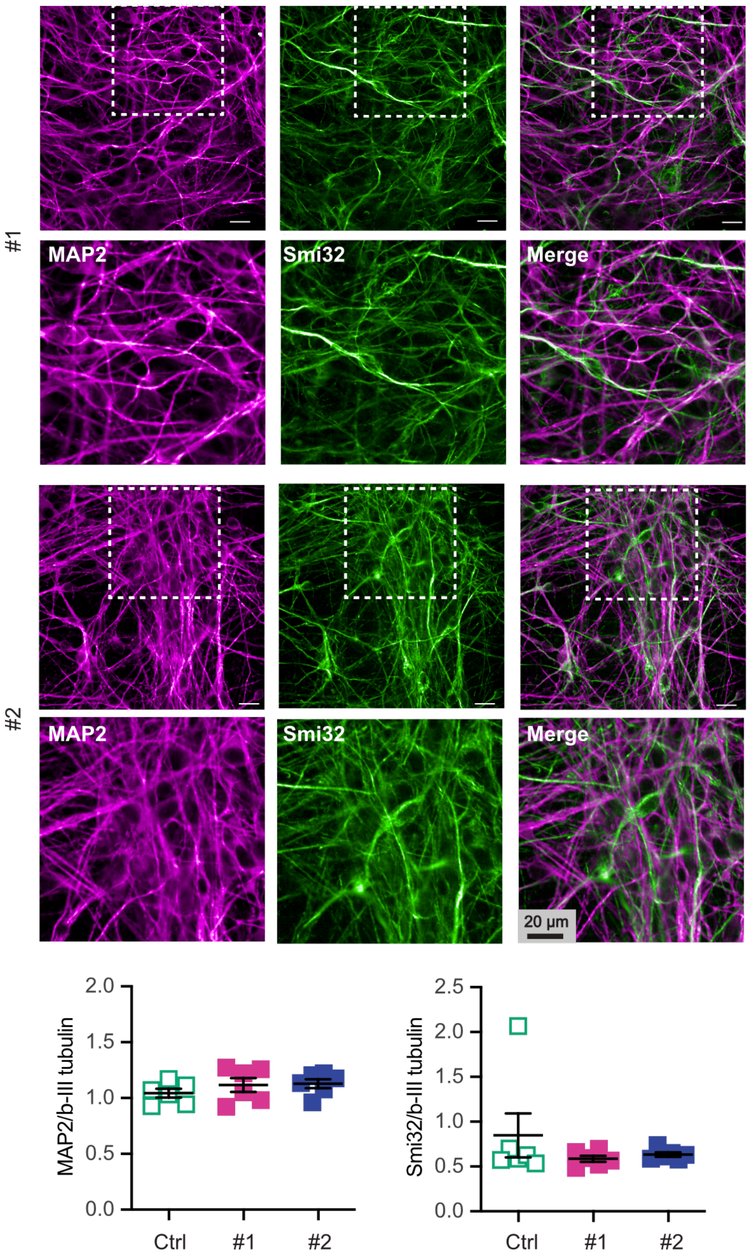

Supplemental Figure 2 Patient-derived motoneurons express similar amounts of MAP2 and Smi32. Immunocytochemical staining of patient-derived motoneurons with Smi32 and MAP2 normalized to the neuronal marker β-tubulin-III (not shown here). Batch analysis calculated the fluorescence intensity of each marker. Data are from three independent differentiation lines performed in triplicates. Statistical variation is shown as scatter plots with the indication of mean ± SEM and significance calculated using the ordinary one-way ANOVA with Turkey´s multiple comparisons test.

Supplemental figure 3

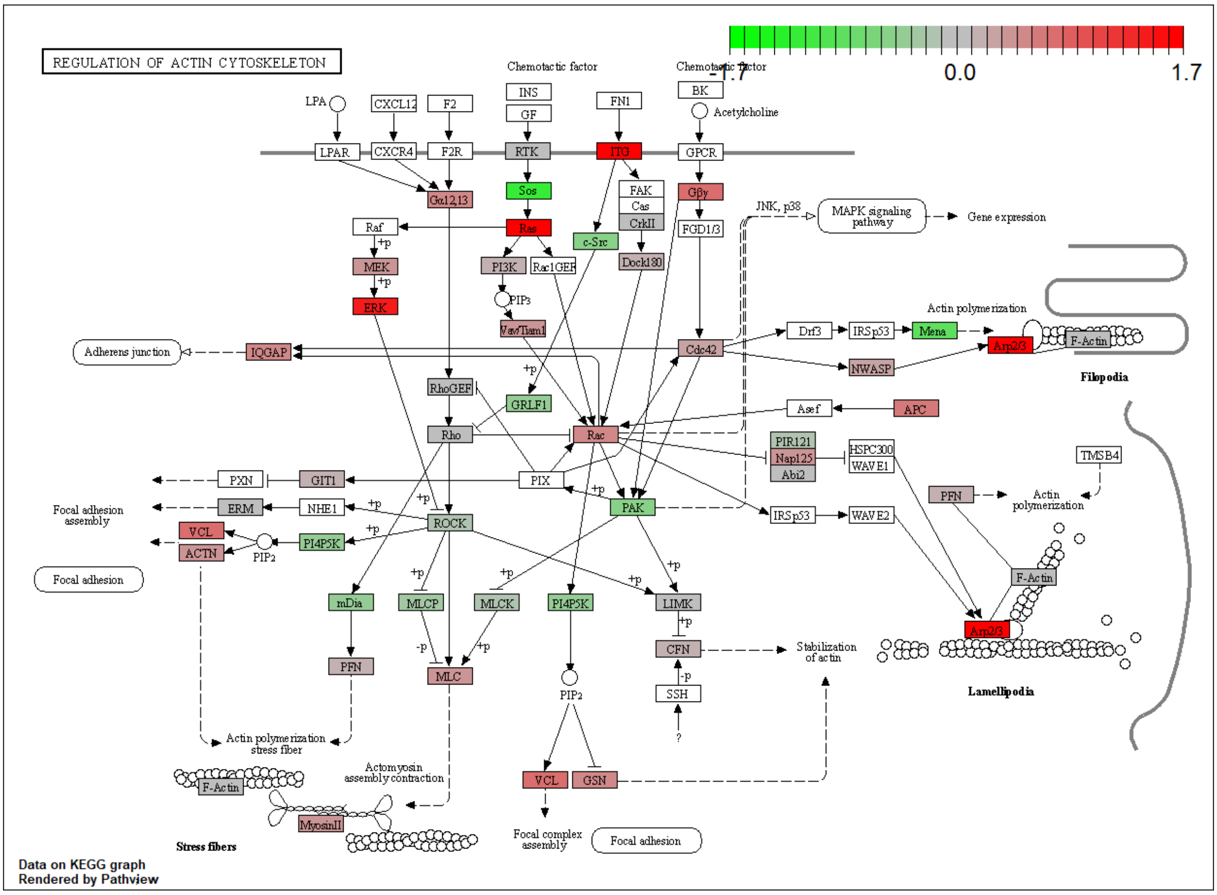

Supplemental Figure 3 Label-free LC-MS proteomics revealed a disruption of actin cytoskeleton regulation (pathway hsa04810) by KEGG analysis due to *GDAP1* knockdown. Proteins are colored in function of their Log2-Fold-Change (Log2FC) between *GDAP1* KD and control cells. Upregulated proteins in *GDAP1* KD are shown in red and downregulated proteins in green. Pathway visualization was performed using the R package clusterProfiler.
