# Supplementary material for: GDAP1 loss of function inhibits the mitochondrial pyruvate dehydrogenase complex by altering the actin cytoskeleton
Source: Commun Biol. 2022 Jun 3;5:541. doi: 10.1038/s42003-022-03487-6 (PMC9166793; doi:10.1038/s42003-022-03487-6)
Supplement: Supplementary file 2 — Description of Additional Supplementary Files [file 42003_2022_3487_MOESM2_ESM.pdf]

Description of Additional Supplementary Files

File name: Supplementary Data 1
Description: Source data for Fig. 1-4.
