# Supplementary material for: GDAP1 loss of function inhibits the mitochondrial pyruvate dehydrogenase complex by altering the actin cytoskeleton
Source: Commun Biol. 2022 Jun 3;5:541. doi: 10.1038/s42003-022-03487-6 (PMC9166793; doi:10.1038/s42003-022-03487-6)
Supplement: Supplementary file 4 — Reporting Summary [file 42003_2022_3487_MOESM4_ESM.pdf]

nature portfolio

COMMSBio-22-0978-T
Corresponding author(s): Axel Methner
Last updated by author(s): Apr 3, 2022

Reporting Summary

Nature Portfolio wishes to improve the reproducibility of the work that we publish. This form provides structure for consistency and transparency in reporting. For further information on Nature Portfolio policies, see our Editorial Policies and the Editorial Policy Checklist.

Statistics

For all statistical analyses, confirm that the following items are present in the figure legend, table legend, main text, or Methods section.

n/a	Confirmed	
☐	☒	The exact sample size (n) for each experimental group/condition, given as a discrete number and unit of measurement
☐	☒	A statement on whether measurements were taken from distinct samples or whether the same sample was measured repeatedly
☐	☒	The statistical test(s) used AND whether they are one- or two-sided<br>*Only common tests should be described solely by name; describe more complex techniques in the Methods section.*
☒	☐	A description of all covariates tested
☐	☒	A description of any assumptions or corrections, such as tests of normality and adjustment for multiple comparisons
☐	☒	A full description of the statistical parameters including central tendency (e.g. means) or other basic estimates (e.g. regression coefficient) AND variation (e.g. standard deviation) or associated estimates of uncertainty (e.g. confidence intervals)
☒	☐	For null hypothesis testing, the test statistic (e.g. F, t, r) with confidence intervals, effect sizes, degrees of freedom and P value noted<br>*Give P values as exact values whenever suitable.*
☒	☐	For Bayesian analysis, information on the choice of priors and Markov chain Monte Carlo settings
☒	☐	For hierarchical and complex designs, identification of the appropriate level for tests and full reporting of outcomes
☒	☐	Estimates of effect sizes (e.g. Cohen's d, Pearson's r), indicating how they were calculated

Our web collection on statistics for biologists contains articles on many of the points above.

Software and code

Policy information about availability of computer code

Data collection	Excel
Data analysis	GraphPad Prism version 9.2

For manuscripts utilizing custom algorithms or software that are central to the research but not yet described in published literature, software must be made available to editors and reviewers. We strongly encourage code deposition in a community repository (e.g. GitHub). See the Nature Portfolio guidelines for submitting code & software for further information.

Data

Policy information about availability of data

All manuscripts must include a data availability statement. This statement should provide the following information, where applicable:

- Accession codes, unique identifiers, or web links for publicly available datasets
- A description of any restrictions on data availability
- For clinical datasets or third party data, please ensure that the statement adheres to our policy

Knockdown vs control proteomics: Project accession: PXD028460, Username: reviewer_pxd028460@ebi.ac.uk, Password: YrBEISO6
Pulldown proteomics: Project accession: PXD024555, Username: reviewer_pxd024555@ebi.ac.uk, Password: uYCQNbDg

Field-specific reporting

Please select the one below that is the best fit for your research. If you are not sure, read the appropriate sections before making your selection.

☒ Life sciences ☐ Behavioural & social sciences ☐ Ecological, evolutionary & environmental sciences

For a reference copy of the document with all sections, see nature.com/documents/nr-reporting-summary-flat.pdf

Life sciences study design

All studies must disclose on these points even when the disclosure is negative.

Sample size	Normal distribution was tested using the D'Agostino-Pearson omnibus normality test. Statistical significance was then verified using appropriate parametric (Student's t test or ANOVA) or non-parametric tests (Mann Whitney and Kruskal Wallis tests) followed by multiple comparison tests as indicated. The Wilcoxon signed rank test was used when normalization to 100 % was necessary as indicated. Statistical analysis of mass spectrometry data was performed using two-tailed, paired t-tests and subsequent Bonferroni correction. Here, a corrected p<0.01 was considered significant for the biotinylated Avi-GDAP1 pull-down experiment and p<0.05 for whole cell lysates. In all other data a p<0.05 was considered to be statistically significant. Pathway over-representation analysis was performed using the STRING database with default parameters 39. KEGG pathway visualization was performed using the R package clusterProfiler 73.
Data exclusions	No data were excluded
Replication	Experiments were reproduced on different days and different passages as indicated
Randomization	Experiments were not randomized
Blinding	Investigators were blinded where possible

Reporting for specific materials, systems and methods

We require information from authors about some types of materials, experimental systems and methods used in many studies. Here, indicate whether each material, system or method listed is relevant to your study. If you are not sure if a list item applies to your research, read the appropriate section before selecting a response.

Materials & experimental systems

n/a	Involved in the study	
☐	☒	Antibodies
☐	☒	Eukaryotic cell lines
☒	☐	Palaeontology and archaeology
☒	☐	Animals and other organisms
☒	☐	Human research participants
☒	☐	Clinical data
☒	☐	Dual use research of concern

Methods

n/a	Involved in the study	
☒	☐	ChIP-seq
☒	☐	Flow cytometry
☒	☐	MRI-based neuroimaging

Antibodies

Antibodies used	Primary antibodies were anti-actin mAB (clone C4, 1:4000; Millipore MAB1501), anti-Cofilin-1 mAB (clone D3F9, 1:1000, Cell Signaling, 5175), anti-p-Cofilin-1 mAB (Ser3, clone 77G2, 1:1000, Cell Signaling 3313), anti-DRP1 mAB (clone 4E11B11, 1:1000, Cell Signaling 14647), anti-GDAP1 (1:750, Sigma HPA014266), anti-G6PD mAB (clone D5D2, 1:1000, Cell Signaling 12263), anti-GAPDH mAB (clone 14C10, 1:2000, Cell Signaling 2118), anti-GLUD1 mAB (clone D9F7P, 1:1000, Cell Signaling 12793), anti-HK1 mAB (clone C35C4, 1:1000; Cell Signaling 2024), anti-HK2 mAB (clone C64G5, 1:1000, Cell Signaling 2687), anti-HSPA9 (clone N52A/42, 1:1000, UC Davis/NIH NeuroMab Facility, Davis, USA, 73-127), anti-LDHA mAb (clone C4B5, 1:1000, Cell Signaling 3582), anti-MCU (1:1000, Sigma HPA016480), anti-MFF (1:1000, Proteintech 17090-1-AP), anti-MFN2 mAB (clone M03, 1:500; Abnova H00009927-M03), anti-Nestin mAB (clone rat-401, 1:1000, Merck chemicals MAB353), anti-PDH mAb (E1α, clone C54G1, 1:1000, Cell Signaling 3205), anti-PDH mAB (E2,E3bp, clone 13G2AE2BH5, 1:1000, Abcam, ab110333) anti-p-PDH (Ser293, 1:1000, Cell Signaling 31866), anti-PKM1/2 mAb (clone C103A3, 1:1000, Cell Signaling 3582), anti-TOM20 (1:1000, Sigma HPA011562), anti-βIII-Tubulin mAB (clone TuJ-1, 1:1000, R&D Systems MAB1195), anti-VDAC1 (clone 20B12AF2, 1:1000, Abcam ab14734).
Validation	Validation was done by relying on manufacturers' information, size of the labeled product and intracellular staining matching the expected pattern.

Eukaryotic cell lines

Policy information about cell lines

Cell line source(s)	The SH-SY5Y cells were a kind gift of David Pla-Martin. The iPSC cell lines were generated by Oliver Brüstle as described in the manuscript.
Authentication	The SH-SY5Y cell lines were not authenticated
Mycoplasma contamination	We regularly test for myoplasma contamination by PCR
Commonly misidentified lines (See ICLAC register)	No commonly misidentified cell lines were used in this study
